# Depression and depressive symptoms in physicians prior to the COVID-19 pandemic: a systematic review and meta-analysis

**DOI:** 10.3389/fpsyt.2025.1627507

**Published:** 2025-10-22

**Authors:** Shan Dhaliwal, Deena Fremont, Wenshan Li, Daniel Myran, Marco Solmi, Peter Tanuseputro, Janet Wilson, Manish M Sood

**Affiliations:** ^1^ The Ottawa Hospital Research Institute, Ottawa, ON, Canada; ^2^ ICES uOttawa, Ottawa, ON, Canada; ^3^ School of Epidemiology and Public Health, University of Ottawa, Ottawa, ON, Canada; ^4^ Department of Family Medicine, University of Ottawa, Ottawa, ON, Canada; ^5^ Department of Psychiatry, Faculty of Medicine, University of Ottawa, Ottawa, ON, Canada; ^6^ Department of Child and Adolescent Psychiatry, Charité Universitätsmedizin, Berlin, Germany; ^7^ Department of Family Medicine and Primary Care, The University of Hong Kong, Hong Kong, Hong Kong SAR, China; ^8^ Faculty of Medicine, University of Ottawa, Ottawa, ON, Canada; ^9^ Department of Medicine, The Ottawa Hospital, Ottawa, ON, Canada

**Keywords:** depression, physicians, depressive symptoms, prevalence, systematic review & meta-analysis

## Abstract

**Background:**

Mental health disorders, such as depression, can significantly impact a physician’s well-being and the quality of care they provide. We conducted a systematic review and meta-analysis to identify risk factors and to estimate the prevalence of depression and depressive symptoms in physicians prior to the COVID-19 pandemic.

**Methods:**

This PRISMA 2020–compliant systematic review and meta-analysis searched EMBASE, APA PsycINFO, and MEDLINE databases for studies published between January 2002 and March 2020 (pre–COVID-19 period). Risk of bias was assessed using a modified Newcastle-Ottawa Scale for cohort and cross-sectional studies. We included studies of physicians where depression/depressive symptoms were measured by either a validated questionnaire or clinical diagnosis. The primary and secondary outcomes measures included assessing the prevalence of depression/depressive symptoms, and whether depression differed by pertinent risk factors (study design, sex, specialty, training stage) in the literature prior to the COVID-19 pandemic.

**Results:**

Forty-two studies from 14 countries involving 27,284 physicians (7,293 with depression or depressive symptoms) were included. The pooled prevalence estimate was 34.2% (95% CI: 26.4-43.0%), with substantial heterogeneity identified across studies (I^2^ = 98%). Most studies were cross-sectional surveys (n=28) and cohort studies (n=14). A total of 13 different assessment methods were used. We found no statistically significant difference in depression between male and female physicians (OR: 0.78, 95% CI: 0.46, 131), and a slightly increased rates in residents compared to staff physicians [pooled estimates of 36% (95% CI: 26-47%) and 29% (95% CI: 13-53%)]. Finally, 25 studies were deemed “High” risk of bias, while the remaining 17 were “Low” risk.

**Conclusions:**

In this review examining depression and depressive symptoms among physicians, we report a pooled estimate of 34% prior to the COVID-19 pandemic. Due to the high degree of heterogeneity in study design and limited examination of key risk factors, limited conclusions can be made regarding the true prevalence across the physicians, and how best to target interventions.

**Systematic review registration:**

https://www.crd.york.ac.uk/prospero/, identifier CRD42021232814.

## 1 Introduction

Prior to the COVID-19 pandemic, mental illness was the leading cause of global health-related burden (1). The onset of the pandemic created an environment in which factors contributing to poor mental health intensified, due to heightened uncertainty surrounding the crisis. Many professionals, including but not limited to frontline physicians, experienced burnout and exhaustion during this stressful time period. While the pandemic amplified these concerns across healthcare professions, physicians represent a unique group given the high intensity demands of clinical care and the psychologically taxing responsibilities of patient care (2, 3). These conditions, which were already prominent in the physician community prior to the pandemic, are the beginning of a continuum that may lead to depressive symptoms and an elevated risk of suicide (4–8). Furthermore, depressive symptoms in physicians not only impact individual well-being, but also have been associated with medical errors, and low quality of patient care (9, 10). Early identification of high-risk individuals may alleviate suffering, improve physician health and well-being, and reduce leaves of absence.

Due to these factors, researchers have been interested in understanding the impact of COVID-19 on mental illness. However, no systematic reviews and meta-analyses to date have assessed the prevalence of physician mental health, specifically depressive symptoms, across all career stages prior to the COVID-19 pandemic. A previous systematic review focusing exclusively on medical residents found that 28.8% reported depressive symptoms (11). However, whether this is consistent after the completion of medical training and/or varies by age, sex, and medical specialty remains unknown. This study advances previous work by comprehensively examining the extent of depressive symptoms in physicians and whether symptoms differ by age, sex, training stage (residents, staff physicians), and specialty, prior to the COVID-19 pandemic. Understanding the pre-pandemic prevalence of depressive symptoms in physicians will provide an essential baseline against which post-pandemic studies can be compared, allowing for a clear assessment of the pandemic’s long-term impact.

## 2 Methods

This systematic review and meta-analysis were developed based on the Preferred Reporting Items for Systematic Review and Meta-Analysis PRISMA-2020 checklist guidelines (**Supplementary Material**). We registered this study PROSPERO International Prospective Register of Systematic Reviews (ID CRD42021232814).

### 2.1 Inclusion/exclusion criteria

Studies of medical doctors, including residents, fellows, and staff physicians from population-based cohort, cross-sectional, and randomized control studies were included. The primary outcome was depression or depressive symptoms, defined by a clinical diagnosis, a validated questionnaire, or physician-administered scale. Definitions of depression and depressive symptoms were determined based on the terminology and criteria used in each included study. We excluded articles i) where depression was assessed by a non-validated measure, ii) case-control studies and iii) medical students, iv) non-physician healthcare professionals, or v) in any language other than English (for reviewer comprehension purposes).

### 2.2 Search strategy

We searched the following databases: Medline, EMBASE, and APA PsycINFO (OVID interface) for articles from January 2002 until March 2020. The study period was selected to reflect contemporary literature in the area and shifts in physician demographics (more females entering the physician workforce). Articles post-March 2020 were excluded to establish the state of physician depression and depressive symptoms prior to the COVID-19 pandemic. Articles from grey literature were also excluded. The search strategy was reviewed by a health science librarian with expertise in systematic reviews (see **Data Supplementary File**).

### 2.3 Study records and data extraction

All relevant articles were reviewed by two independent screeners (AF and SD) for inclusion and categorized as eligible, ineligible, or possibly eligible. Any conflicts were reviewed by two independent authors (ER and NC). Articles marked as ‘Yes’ had their full texts retrieved and reviewed for final inclusion (based on consensus between SD and HD). Following full-text screening, two reviewers (HD and SD) independently extracted data from included full-text articles and entered findings into a data extraction form. Originally, our study aimed to study the available literature on MDD; however, it was found that most studies focused on depression and depressive symptoms. Thus, a decision was made to focus instead on the latter.

The following information was extracted: descriptive statistics related to study location, year of publication, study design, age and sex of participants, and level of training; number of individuals who were assessed for depression or depressive symptoms; the number of individuals who screened positive for depression or depressive symptoms; any subgroup assessments where the results were stratified by sex, age, or level of training; the method of assessment; and cut-off scores for assessing depression or depressive symptoms. No assumptions were made regarding missing data. After data extraction, data extraction results were compared between reviewers and consolidated into a final data sheet used for further analysis. Conflicts at this stage were resolved through discussion between the reviewers (SD and HD) responsible for extraction. The Covidence Systematic Review Management Software (Covidence, Melbourne, VIC, Australia) was used for screening (12).

### 2.4 Data synthesis

Categorization of depression and/or depressive symptoms was heterogeneous in our screened articles. For the purposes of meta-analyses, results of each study were treated as dichotomous variables, with any instance of depression or depressive symptoms considered as a positive screen. Pooled results were calculated for all included studies and subgroups of study design, sex, specialty, and training period. In studies examining the same study population, only the study with the largest sample size was included. Random-effects modelling was utilized to estimate a pooled prevalence of depression or depressive symptoms based on the proportions reported by each study. From these proportions, the Clopper-Pearson method was used to derive confidence intervals (13). For the purposes of calculating pooled odds ratio estimates, the Mantel-Haenszel method was used to determine the weight of each study and Knapp-Hartung adjustments were made for the random effects model (14). In the case of prospective and retrospective cohort studies that reported proportional estimates made throughout the course of the study, an overall period proportion was used. Heterogeneity was assessed by the I^1^ statistic and X^2^ tests. In addition, a single-study exclusion sensitivity analysis was done to estimate the effect of each study on the pooled estimate. Results were presented in the form of forest plots. All analyses were done following Harrer et al.’s guide to Meta-Analysis and conducted using R version 4.2.2 (R Foundation for Statistical Computing) with RStudio (13, 15, 16). All statistical tests were 2-sided and used a threshold of p<0.05.

### 2.5 Risk of bias assessment

To assess the quality of studies included in this review, the modified version of the Newcastle-Ottawa Scale (NOS) was used for both cohort and cross-sectional studies (17). NOS includes sections to assess the selection, comparability with the general population, and assessment of the outcome. The forms for the modified NOS are included in the **Data Supplementary File**.

## 3 Results

### 3.1 Study characteristics

**Figure 1:**
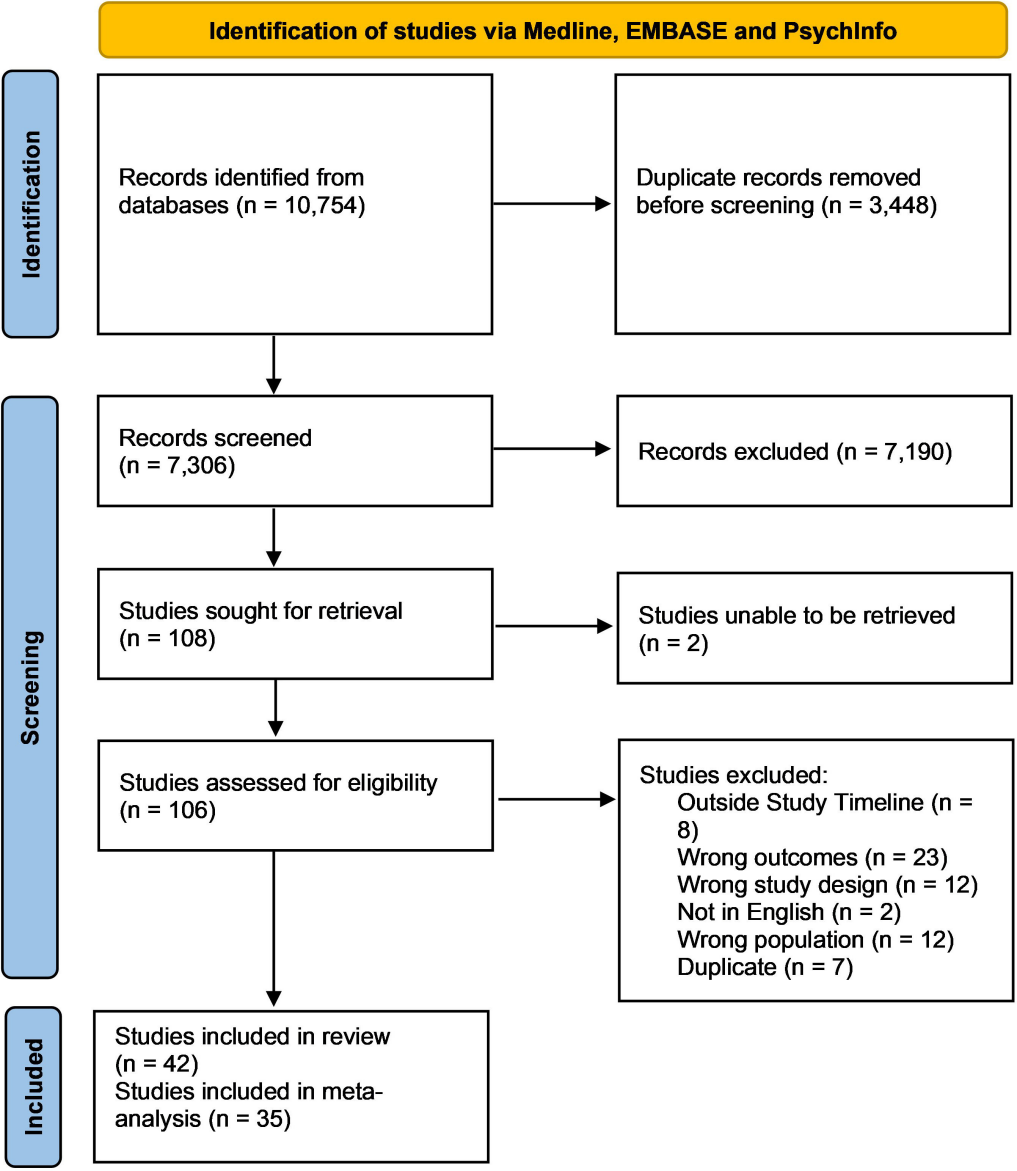
PRISMA flow chart.

**Figure 2:**
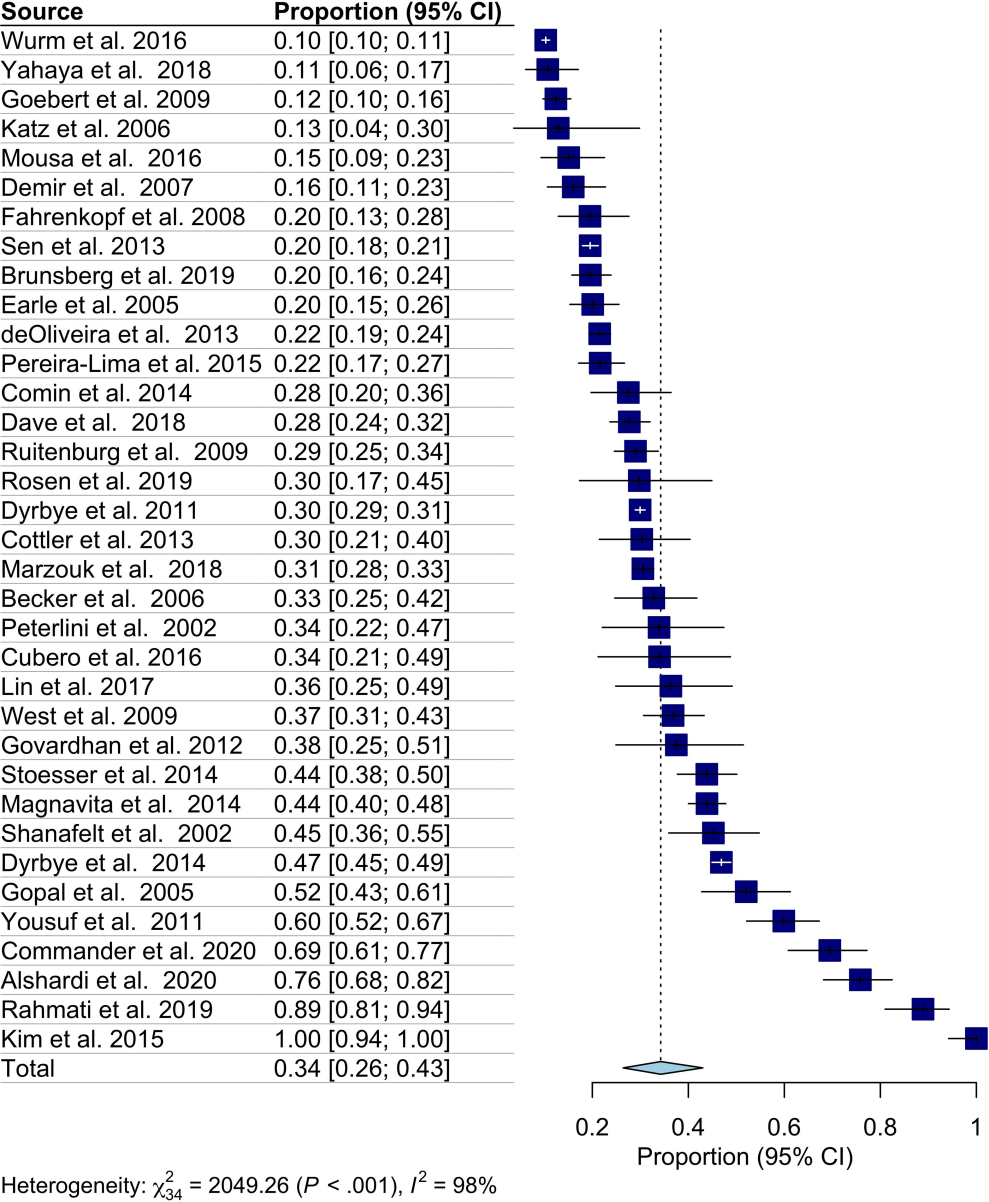
Forest plot on the overall prevalence of depressive symptoms.

A total of 10,754 studies were identified using the search strategy, with 7,306 abstracts being screened out after the removal of duplicate articles. From there, 106 full-text articles were assessed for inclusion, of which 64 articles were removed based on exclusion criteria (**Figure 1**). Overall, 42 studies were included in this systematic review, with 35 of these studies being utilized during meta analyses. Included studies were published between 2002–2020 with the majority being from the United States, and the remaining from Europe, Africa, South America, Asia, and the Middle East. Most studies were cross-sectional (n=28), with the remainder being cohort studies (n=14), which were primarily prospective cohort studies (n=13) and one retrospective cohort study. Overall, using 35 studies encompassing 257,284 physicians with 7,293 screening positive, the pooled estimated proportion of depression and depressive symptoms was 34.2% (CI: 26.3-43.0%) with significant heterogeneity (I^2^ = 98%) (**Table 1**, **Figure 2**). No difference in the pooled estimates was noted with stratification by study design (cross sectional surveys vs. cohort studies, **Supplementary Figure 1**).

**Table 1:** Study characteristics of the design, assessment methods and outcomes for included studies investigating depression and pressive symptoms in physician and resident populations.

Author, year	Location	Study design/MD type	Sample (n)	Response rate (%)	Outcome assessment	Definition of outcome	Outcome n (%)
Balch et al. (18) 2011*	USA	CSS/surgeons	7861 (missing n=63)	35.0	PRIME-MD	Positive Screening	2333 (29.6)
Balch et al. (19) 2010 *	USA	CSS/surgeon	7697 (missing n=53)	32.0	PRIME-MD	Positive Screening	2320 (30.1)
Campbell et al. (20) 2010*	USA	CSS/IM residents	86	48.0	PRIME-MD	Score≥3=Depression	As First Year: 45 (52); Second Year: 41 (48); Third Year: 23 (27)
Dyrbye et al. (21) 2011	USA	CSS/surgeons	7858	31.5	PRIME-MD	Positive Screening	2354 (30.0)
Dyrbye et al. (22) 2012 ^	USA	CSS/surgeons	6240	28.7	PRIME-MD	Positive Screening	2510 (40.2)
Dyrbye et al. (23) 2014	USA	CSS/residents and physicians	2581	22.5	PRIME-MD	Positive Screening	1210 (46.9)
West et al. (24) 2006 *	USA	PCS/IM residents	149	84.0	PRIME-MD	Positive Screening	47 (31.5)
West et al. (25) 2009	USA	PCS/IM residents	239	88.3	PRIME-MD	Positive Screening	88 (36.8)
Gopal et al. (26) 2005	USA	CSS/IM residents a	121	80.5	PRIME-MD	Positive Screening	67 (55.4)
Shanafelt et al. (27)2002	USA	CSS/IM residents	115	76.0	PRIME-MD	Positive Screening	52 (45.2)
Cubero et al. (28) 2016	Brazil	PCS/Oncology Fellows	50	100	BDI	0-4=No depression; 5-7=Mild depression; 8-15=Moderate depression; ≥16=Severe depression	17 (18.8)
Demir et al. (29) 2007	Turkey	CSS/residents	156	75.0	BDI	Score≥17=probable depression	25 (16.0)
Peterlini et al. (30) 2002	Brazil	CSS/residents	59	N/A	BDI	<16=Low depressive symptoms; 16-21=medium depressive symptoms; >21=high depressive symptoms	Total: 20 (33.4); Low: 16 (27.1); Medium: 3 (4.2); High: 1 (2.1)
Kim et al. (31) 2015	South Korea	CSS/residents	62	N/A	BDI	Score >=16 Moderate or Severe Depression	Total: 62 (100.0); Moderate: 10 (16.1) Severe: 52 (83.9)
Lin et al. (32) 2017	USA	PCS/general surgery residents	66	63.0	BDI	0-4=No depression; 5-7=Mild depression; 8-15=Moderate depression; ≥16=Severe depression	Total: 24 (36.4); Mild: 11 (16.7); Moderate: 8 (12.1); Severe: 5 (7.6)
Rosen et al. (33) 2019	US	PCS/residents	47	80.0	BDI	Moderate Depression: Score >= 8	Beginning of residency: 2 (4.3) End of first year: 14 (29.8)
Ruitenburg et al. (34) 2012	Netherlands	CSS	400	51.0	BDI	High likelihood of Depression: Score >= 4	116 (29.0)
Govardhan et al. (35) 2012	USA	CSS/OB/GYN residents	56	46.0	CES	Score≥16=Depressed	21 (37.5)
Goebert et al. (36) 2009	USA	CSS/residents	532	64.0	CES	<16=No depression; 16-21=mild to moderate depression; >21=major depression	Total: 63 (11.9); Minor/Moderate: 25 (4.7) Major: 38 (7.2)
Katz et al. (37) 2006	USA	PCS/Emergency Medicine residents	31	62.0	CES	<16=No depression; 16-21=mild to moderate depression; >21=major depression	4 (12.9)
Becker et al. (38) 2006	USA	CSS/Obstetrics and Gynecology residents	125	29.0	CES	Score≥16=Depressed	41 (32.8)
Yousuf et al. (39) 2011	Pakistan	CSS/residents	172	82.7	Zung Self-rating Depression Scale	N/A	103 (59.9)
Comin et al. (40) 2014	Spain	RCS	120	N/A	Clinical Interview	Positive Screening	33 (27.5)
Yahaya et al. (41) 2018	Malaysia	CSS/Emergency Medicine physicians	140	N/A	DASS-21	Score>13=depression	15 (10.7)
Dave et al. (42) 2018	India	CSS/residents	462	88.8	DASS-21	Normal, mild, moderate, severe, and extremely severe (no scores associated with it)	128 (27.7)
Magnavita et al. (43) 2014	Italy	CSS/Radiologists	654	N/A	Goldberg’s Anxiety and Depression Scales	Score≥4=depression	287 (43.9)
Brunsberg et al. (44) 2019	USA and Canada	PCS/Pediatric Residents	388	72.0	HANDS	Score≥9=Depressed	76 (19.6)
Fahrenkopf et al. (45) 2008	USA	PCS/residents	123	50.0	HANDS	Score≥9=Depressed	24 (19.5)
deOliveira et al. (46) 2013	USA	CSS/anesthesiology residents	1384	54.0	HANDS	Score≥9=Depressed	298 (21.5)
Marzouk et al. (47) 2018	Tunisia	CSS/residents	1700	77.0	HAD	Score 8-10=probable depression; score ≥11=depression	519 (30.5)
Wurm et al. (48) 2016	Austria	CSS	5897	15.8	MDI	Mild: 20-24; Moderate: 25-29; Severe: 30+	Total: 607 (10.4); Mild: 50 (0.9); Moderate: 135 (2.3); Severe: 422 (7.2)
Rahmati et al. (49) 2019	Iran	PCS/Emergency Medicine residents	99	100.0	Minnesota Multiphasic Personality Inventory	Mild/Moderate/Severe/Very severe depression	First year: Total: 88 (88.8); Mild: 34(33.3); Moderate: 42 (42.2); Severe: 12 (12.1) Third Year: Total: 74 (74.7); Mild: 27 (27.3); Moderate: 38 (38.4); Severe: 9 (9.1)
Cottler et al. (50) 2013	USA	PCS	99	N/A	National Epidemiologic Survey on Alcohol and Related Condition	N/A	30 (30.3)
Mousa et al. (51) 2016	USA	CSS/residents	126	24.4	PHQ-2	Score≥3=MDD	19 (15.1)
Pereira-Lima et al. (52) 2015	Brazil	CSS/residents	305	76.25	PHQ-4	Score≥3=Depression	66 (21.6)
Alshardi et al. (53) 2020	Saudi Arabia	CSS/residents	149	72.8	PHQ-9	0-4=No depression; 5-9=Mild depression; 10-14=Moderate depression; 15-19=Moderately severe depression; ≥20=Severe depression	113 (75.8)
Commander et al. (54) 2020	Africa	CSS/surgeons	131	12.4	PHQ-9	Positive screening for depression: mild, moderate, moderately severe, and severe	91 (69.5)
Kalmbach et al. (55) 2017	USA	PCS/residents	933		PHQ-9	Positive screening at 3 or 6 months	151 (12.4)
Sen et al. (56)2010	USA	PCS/residents	740	58.0	PHQ-9	Moderate Depression: 10-14 Moderately Severe: 15-19 Severe Depression: 20+	190 (25.7)
Sen et al. (57) 2013	USA	PCS/IM residents	2323	58.0	PHQ-9	Positive Screening	454 (19.5)
Stoesser et al. (58) 2014	USA	CSS/residents	260	36.9	PHQ-9	0-4=No depression; 5-9=Mild depression; 10-14=Moderate depression; 15-19=Moderately severe depression; 20-27=Severe depression	114 (43.9)
Earle et al. (59) 2005	Canada	CSS/family medicine residents	254	46	Modified PHQ using DSM-IV criteria for depression and anxiety symptoms	N/A	51 (20.0)

CSS, Cross-sectional survey; PCS, prospective cohort study; RCS, Retrospective cohort study; IM, Internal Medicine; PRIME-MD, Primary Care Evaluation of Mental Disorders; BDI, Beck Depression Inventory; PHQ, Patient Health Questionnaire; CES, Center for Epidemiologic Studies Depression Scale; HANDS, Harvard Department of Psychiatry National Depression Screening Day Scale; HAD, Hospital Anxiety and Depression Scale; DASS-21, Depression Anxiety Stress Scales-21; MDD, Major Depressive Disorder; MDI, Major Depression Inventory; N/A, Not Available. *Excluded for the purposes of Meta-Analysis as another study had larger sample size from the same population. ^Excluded from Meta-Analysis except where the favored study did not provide sufficient information for subgroup analysis on sex.

The most common method of depression assessment were the Primary Care Evaluation of Mental Disorders (PRIME-MD) with 10 studies (18–27), followed by the Patient Health Questionnaire (PHQ), and its variations, with nine studies (51–59). The Beck Depression Inventory (BDI) survey was used in seven studies (28–34), while the Center for Epidemiologic Studies (CES) Depression Scale was used 4 times (35–38), the Harvard Department of Psychiatry National Depression Screening Day Scale (HANDS) was used in 3 studies (44–46), and the Depression Anxiety Stress Scales-21 (DASS-21) was used in two studies (39, 40). Finally, the Zung Self-Rating Depression Scale, the National Epidemiologic Survey on Alcohol and Related Conditions, the Minnesota Multiphasic Personality Inventory, the Hospital Anxiety and Depression Scale (HAD), the Goldberg’s Anxiety and Depression Scales, the Major Depression Inventory (MDI), and a clinical interview were used once in the remaining studies (41–43, 47–50). Heterogeneity was observed for defining depression or depressive systems within the same method of assessment. For example, Demir et al. classified probable depression with a score of 17, while Lin et al. defined 5–8 as mild depression, 8–15 as moderate, and greater than 16 as severe depression (29, 32). The studies utilizing the BDI instrument reported 4 different cut-off scores for depression, while those using the CES used a score of 16 or greater to define depression. Similar differences were noted in studies using the Depression and Anxiety Scales (DASS-21), with one study not reporting the cut-off scores but instead only the various categories of depression. Studies reporting the HANDS instrument consistently used the same cut-off score, while the PHQ and its derivatives varied.

### 3.2 Studies by specialty

Twenty studies included participant-level data on medical specialties. Internal medicine (n=6) (20, 24–27, 57) and surgery (n=6) (18, 19, 21, 22, 32, 54) had the greatest number of studies, followed by emergency medicine (n = 3) (31, 38, 42), ‘other’ specialties (n=3), and pediatrics (n=2) (44, 45). The reported proportion of depression or depressive symptoms among surgeons varied between 30-70.5 (18, 19, 32, 54). A similar trend was found for emergency medicine physicians, with two studies showing lower levels of depression (10.7-12.1%), and one reporting much higher prevalences (88.8%) (37, 40, 48). Five of the six studies on internal medicine residents reported relatively high proportions of depression (ranging from 29.8-47.0%), whereas the remaining study found a lower prevalence (13.6%) (20, 24–26, 30, 33). Isolated studies of oncologists and general practitioners reported 34% and 21.5% affected by depression or depressive symptoms, respectively (28, 59). Pediatricians and obstetrics/gynecology each had two studies conducted with 19.5-19.6% and 32.8-37.5% of physicians reporting depression and depressive symptoms, respectively (35, 38, 44, 45). In addition, 21.5% of participating anesthesiologists (n=1 study) and 43.9% of participating radiologists (n=1 study) were reported to have depression or depressive symptoms (43, 46). Only one study stratified depression by different specialties. This study, completed by Balch et al., found that oncologic surgeons reported lower levels of depression relative to other surgeons (18) (**Table 2**).

**Table 2:** Studies reporting depression and depressive symptoms in physicians and residents by specialty and stage of training.

Author, year	Outcome assessment	Definition of outcome	Specialty distribution	Outcome	Training stage distribution	Outcome
Katz et al. (37) 2006	CES	<16=No depression; 16-21=mild to moderate depression; >21=major depression	Emergency: 31	Emergency: 4 (12.9)	Residents: 31	4 (12.9)
Mousa et al. (51) 2016	PHQ-2	Score≥3=MDD	N/A	N/A	Residents: 126	19 (15.1)
Demir et al. (29) 2007	BDI	Score≥17=probable depression	N/A	N/A	Residents: 156	25 (16.0)
Stoesser et al. (58) 2014	PHQ-9	Score≥10=Depression	N/A	N/A	Residents: 260	114 (43.8)
Fahrenkopf et al. (45) 2008	HANDS	Score≥9=Depressed	Pediatrics: 123	Pediatrics: 24 (19.5)	Residents: 123	24 (19.5)
Sen et al. (57) 2013	PHQ-9	Positive Screening	N/A	N/A	Residents: 2323	454 (19.5)
Brunsberg et al. (44) 2019	HANDS	Score≥9=Depressed	Pediatrics: 388	Pediatrics: 76 (19.6)	Residents: 388	76 (19.6)
deOliveira et al. (46) 2013	HANDS	Score≥9=Depressed	Anesthesiology: 1384	Anesthesiology: 298 (21.5)	Residents: 1384	298 (21.5)
Pereira-Lima et al. (52) 2015	PHQ-4	Score≥3=Depression	N/A	N/A	Residents: 305	66 (21.6)
Dave et al. (42) 2018	DASS-21	Normal, mild, moderate, severe, and extremely severe (no scores associated with it)	N/A	N/A	Residents: 462	128 (27.7)
Marzouk et al. (47) 2018	HAD	Score 8-10=probable depression; score ≥11=depression	N/A	N/A	Residents: 1700	519 (30.5)
Becker et al. (38) 2006	CES	Score≥16=Depressed	Obstetrics and Gynecology: 125	Obstetrics and Gynecology: 41 (32.8)	Residents: 125	41 (32.8)
Shanafelt et al. (27) 2002	PRIME-MD	Positive Screening	N/A	N/A	Residents: 115	52 (45.2)
Lin et al. (32) 2017	BDI	0-4=No depression; 5-7=Mild depression; 8-15=Moderate depression; ≥16=Severe depression	Surgeons: 66	Surgeons: 24 (36.4)	Residents: 66	24 (36.4)
West et al. (25) 2009	PRIME-MD	Positive Screening	Internal Medicine: 239	Internal Medicine: 88 (36.8)	Residents: 239	88 (36.8)
Govardhan et al. (35) 2012	CES	Score≥16=Depressed	Obstetrics and Gynecology: 56	Obstetrics and Gynecology: 21 (37.5)	Residents: 56	21 (37.5)
Kim et al. (31) 2015	BDI	Score >= Moderate or Severe Depression	N/A	N/A	Residents: 62	62 (100.0)
Gopal et al. (26) 2005	PRIME-MD	Positive Screening	Internal Medicine: 227	Internal Medicine: 105 (46.3)	Residents: 121	67 (55.4)
West et al. (24) 2006 *	PRIME-MD	Positive Screening	Internal Medicine: 149	Internal Medicine: 70 (47.0)	Residents: 149	47 (31.5)
Yousuf et al. (39) 2011	Zung Self-rating Depression Scale	N/A	N/A	N/A	Residents: 172	103 (59.9)
Alshardi et al. (53) 2020	PHQ-9	0-4=No depression; 5-9=Mild depression; 10-14=Moderate depression; 15-19=Moderately severe depression; ≥20=Severe depression	N/A	N/A	Residents: 149	113 (75.8)
Sen et al. (56) 2010	PHQ-9	Moderate Depression: 10-14 Moderately Severe: 15-19 Severe Depression: 20+	N/A	N/A	Residents: 740	190 (25.7)
Peterlini et al. (30) 2002	BDI	<16=Low depressive symptoms; 16-21=medium depressive symptoms; >21=high depressive symptoms	Internal Medicine: 59	Internal Medicine: 8 (13.6)	Residents: 59	20 (33.9)
Kalmbach et al. (55) 2017	PHQ-9	Positive screening at 3 or 6 months	N/A	N/A	Residents: 933	115 (9.5)
Earle et al. (59) 2005	Modified PHQ using DSM-IV criteria for depression and anxiety symptoms	N/A	Family Medicine: 254	Family Medicine: 51 (20.0)	Residents: 254	51 (20.0)
Cubero et al. (28) 2016	BDI	0-4=No depression; 5-7=Mild depression; 8-15=Moderate depression; ≥16=Severe depression	Oncology: 50	Oncology: 17 (34.0)	Residents: 50	17 (34.0)
Campbell et al. (20) 2010	PRIME-MD	Score≥3=Depression	Internal Medicine: 258	Internal Medicine: 109 (42.3)	Residents: 86	As First Year: 45 (52); Second Year: 41 (48); Third Year: 23 (27)
Rahmati et al. (49) 2019	Minnesota Multiphasic Personality Inventory	Mild/Moderate/Severe/Very severe depression	Emergency: 99	As First Year: 88 (88.8) As Third Year: 74 (74.7)	Residents: 99	As First Year: 88 (88.8) As Third Year: 74 (74.7)
Goebert et al. (36) 2009	CES	<16=No depression; 16-21=mild to moderate depression; >21=major depression	N/A	N/A	Residents: 532; PGY1: 220; PGY2: 84; PGY3: 96; PGY4: 62; PGY5+: 70	Total: 66 (12.4) ^&;^ PGY1: 24 (10.9); PGY2: 12(14.3); PGY3: 14 (14.6); PGY4: 11 (17.8) PGY5+: 5 (7.2)
Wurm et al. (48) 2016	MDI	Mild: 20-24 Moderate: 25-29 Severe: 30+	N/A	N/A	Staff Physician: 5897	607 (10.3)
Yahaya et al. (41) 2018	Score>13=depression	30 (10.7)	Emergency: 140	Emergency: 30 (10.7)	Staff Physician: 140	15 (10.7)
Balch et al. (18) 2011	PRIME-MD	Positive Screening	Oncologic Surgeons: 407 Other Surgeons: 7454	Oncologic: 99 (24.3) Other Surgeons: 2234 (30.2)	Staff Physician: 7861 (missing n=63)	2333 (29.6)
Balch et al. (19) 2010	PRIME-MD	Positive Screening	Surgeons: 7697	Surgeons: 2320 (30.1)	Staff Physician: 7748 (missing n=53)	2332 (30.0)
Dyrbye et al. (21) 2011	PRIME-MD	Positive Screening	Surgeons: 7858	Surgeons: 2354 (30.0)	Staff Physician: 7858	2354 (30.0)
Dyrbye et al. (22) 2012	PRIME-MD	Positive Screening	Surgeons: 6240	Surgeons: 2510 (40.2)	Staff Physicians: 6240	2510 (40.2)
Commander et al. (54) 2020	PHQ-9	Positive screening for depression: mild, moderate, moderately severe, and severe	Surgeons: 129	Surgeons: 91 (70.5)	Staff Physician: 131	91 (94.8)
Ruitenburg et al. (34) 2012	BDI	High likelihood of Depression: Score >= 4	N/A	N/A	Residents: 184 Staff Physicians: 216	Residents: 57 (31.0) Staff Physicians: 58 (27.0)
Dyrbye et al. (23) 2014	PRIME-MD	Positive Screening	N/A	N/A	Residents: 1701 Early Career Physicians: 880	Residents: 861 (50.6) Early Career Physicians: 349 (39.7)
Rosen et al. (33) 2006	BDI	Moderate Depression: Score >= 8	Internal Medicine: 47	Beginning of residency: 2 (4.3) End of first year: 14 (29.8)	Residents: 47	Beginning of residency: 2 (4.3) End of first year: 14 (29.8)
Magnavita et al. (43) 2014	Goldberg’s Anxiety and Depression Scales	Score≥4=depression	Radiology: 654	Radiology: 298 (21.5)	Staff: 654	Staff: 298 (21.5)

CSS, Cross-sectional survey; PCS, prospective cohort study; RCS, Retrospective cohort study; PRIME-MD, Primary Care Evaluation of Mental Disorders; BDI, Beck Depression Inventory; PHQ, Patient Health Questionnaire; CES, Center for Epidemiologic Studies Depression Scale; HANDS, Harvard Department of Psychiatry National Depression Screening Day Scale; HAD, Hospital Anxiety and Depression Scale; DASS-21, Depression Anxiety Stress Scales-21; MDD, Major Depressive Disorder; MDI, Major Depression Inventory. *Excluded for the purposes of Meta-Analysis as another study had larger sample size from the same population. N/A, Not Available. ^&^Due to issues with significant figure rounding, this estimated prevalence differed from that of expected in **Table 1** (for the purposes of Meta-Analysis, the results from **Table 1** used to ensure consistency).

After removing duplicate studies, surgeons (n=3) were found to have the highest overall pooled estimate for depression at 45% (95% CI: 26-66%) with a high degree of heterogeneity (*I* ^2^ = 98%) (**Supplementary Figure 2**). This was then followed by radiology (n=1), with a pooled prevalence of 44% (95% CI: 40-48%), and internal medicine (n=4), with a pooled prevalence of 39% (95% CI: 31-47%. The lowest prevalence of depression was seen in family medicine and pediatrics, with pooled estimates of 20% (95% CI:15-26%) and 20% (95% CI:16-23%), respectively.

### 3.3 Studies by sex

Six studies stratified rates of depression or depressive symptoms by sex. Overall, a slightly higher proportion of female physicians screened positive for depression or depressive symptoms (548/1634, 33.5%), compared to their male colleagues (2165/7319, 29.6%). Furthermore, male physicians were less likely to report depressive symptoms compared to female physicians (OR: 0.78, 95% CI: 0.46; 1.31, *I* ^2^ = 37%) (**Supplementary Figure 3**). However, these results were not consistent. Yahaya et al. found that female physicians had a lower prevalence of depression (8.3%), compared to male physicians (10.7% - **Table 3**).

**Table 3:** Studies reporting depression and depressive symptoms in physicians and residents by age and sex.

Author, year	Location	Sex n (%) male	Outcome assessment	Definition of outcome	Outcome	Age distribution n (%)	Outcome
Dyrbye et al. (60) 2011	USA	6815 (86.7)	PRIME-MD	Positive Screening	M: 2010 (29.5) F: 344 (33)	N/A	N/A
Yahaya et al. (41) 2018	Malaysia	56 (40.0)	DASS-21	Score>13=depression	M: 8 (14.3) F: 7 (8.3)	20-29: 62 30-39: 73 >=40: 5	20-29: 4 (6.2) 30-39: 11 (15.1) >=40: 0
Stoesser et al. (58) 2014	Utah, USA	126 (50.2)	PHQ-9	Score≥10=Depression	M: 45 (35.7) F: 47 (37.6)		
Ruitenburg et al. (34) 2012	Netherlands	189 (47.4)	BDI	High likelihood of Depression: Score >= 4	M: 47 (25.0) F: 67 (32.0)	20-35: 170 36-45: 116 46-55: 64 >=56: 50	20-35: 49 (29.0) 36-45: 31 (27.0) 46-55: 19 (30.0) >=56: 17 (34.0)
Alshardi et al. (53) 2020	Jedah, Saudi Arabia	70 (47.0)	PHQ-9	0-4=No depression; 5-9=Mild depression; 10-14=Moderate depression; 15-19=Moderately severe depression; ≥20=Severe depression	M: 51 (72.9) F: 62 (78.5)	<=26: 76 >26: 73	<=26: 55 (72.4) >26: 53 (72.9)
Mousa et al. (51) 2016	New York, USA		PHQ-2	Score≥3=MDD	N/A	18-24: 158 25-30: 233 31-35: 53 36-45: 18	18-24: 29 (18.4) 25-30: 34 (14.6) 31-35: 9 (17.0) 36-45: 2 (11.1)
Demir et al. (29) 2007	Istanbul, Turkey	63 (40.4)	BDI	Score≥17=probable depression	M: 4 (6.3) W: 21 (22.6)	<29: 122 >30: 34	<29: 19 (15.6) >30: 6 (17.6)

abbrev, meaning; *Insufficient/incomplete data to calculate and include in meta-analysis.

### 3.4 Studies by level of training

Of the included studies, 30 reported on residents as the study population (25 were included for meta-analysis due to duplicate study populations, with eight including the year of training of included residents), nine included fully trained staff physicians (five were used in meta-analysis), and two included information on both residents and physicians (**Supplementary Figures 4**, **5**). One study that focused on fellows by Cubero et al. (included in resident population) reported that 34.0% of medical oncology fellows were depressed (28). There was large variability in the 28 studies reporting on depression in residents (6% to 100%). This same large range in depression was observed across all physician training levels (10% to 70.5%; **Table 2**). Three studies reported depression to decrease as physicians went through training, while one study reported the opposite trend. The overall pooled estimate in studies of residents was slightly higher at 36% (95% CI: 26-47%, *I* ^2^ = 96%) compared to 29% (95% CI: 13-53%, *I* ^2^ = 100%) in physicians (**Supplementary Figures 4**, **5**). However, in the two studies reporting both physicians and residents, no difference was noted (OR: 0.66, 95% CI: 0.22;1.98, *I* ^2^ = 5%) (**Supplementary Figure 6**).

### 3.5 Studies by age

Five studies reported depression physicians age. Four studies reported higher levels of depression in older physicians (after combining the 30–39 and >=40 age categories given by Yahaya et al.) (29, 34, 40, 53). However, Ruitenburg et al. found lower levels of depression in younger (20–35-year-old doctors) and older (>=56 years old) physicians than compared to their colleagues aged 36-55 (34). Conversely, Mousa et al. reported lower levels of depression among older physicians compared to younger, with variability between age categories (51) (**Table 3**). Due to the large heterogeneity between the included studies and the lack of individual participant data being reported, a meta-analysis was not conducted.

### 3.6 Risk of bias assessment

A modified NOS was used for assessing the quality of both the included cohort and cross-sectional studies (**Supplementary Table 1**). Overall, 25 studies were marked as having a “High” risk of bias, while 17 were marked as being “Low” risk of bias. This was largely due to many studies being conducted at a single institution, within a single specialty, or with too small a sample size. Furthermore, many studies used self-reported data, which increases the risk of bias.

## 4 Discussion

This systematic review and meta-analysis included 42 studies encompassing data on more than 50,000 physicians. Variability in study design was observed, with studies predominately being cross-sectional surveys (n=28), prospective cohort (n=13), and retrospective cohort studies (n=1), with a high degree of heterogeneity in methods to assess depression. We found a pooled proportional estimate of 34.2% (95% CI: 26.4-43.0%, 35 studies) among the reported literature. Few studies reported key physician characteristics or provided direct comparisons that could aid in the potential identification of susceptible populations. Among specialties, surgeons reported the highest proportion of depression/depressive symptoms, followed by emergency and internal medicine physicians. No significant differences in depression were identified across physician age groups or by sex.

The studies included in this review utilized 13 different assessment methods, including but not limited to the PRIME-MD, BDI, and variations of the PHQ. While these methods have been validated for assessing depression and depressive symptoms, different cut-off scores were used between the studies observed. For example, Demir et al. used the BDI with a cut-off score of at least 16 to state than an individual has probable depression, while Lin et al. stated that a score between 5–8 indicated mild depression; 8–15 indicated moderate; and score greater than 16 indicated severe depression despite using the same assessment method (29, 32). These discrepancies in cut-off score limit the generalizability of each study and inadvertently may exclude individuals with milder depression/depressive symptoms. In addition, as highlighted by Mata et al., some of these instruments have low specificity and therefore are more commonly used as screening tools rather than clinician-administered assessments (11). Moving forward, the adoption of a single assessment method of screening with an established and strict cut-off score in future studies would facilitate inter-study comparisons.

We report an estimated pooled proportion of 34.2% (95% CI: 26.4-43.0%), with all the meta-analyzed studies finding more than 10% of included individuals screened positive. This estimate is higher than the prevalence previously reported by Mata et al. among residents (28.8%) (7),suggesting that studies published between 2015 and 2020 report a higher prevalence of depression in physicians compared with studies conducted between 2002 and 2015. Our pooled proportion is higher than the estimated 4.4% of the global population being affected with depression and may reflect a true higher prevalence among physicians or the inclusion of less severe depressive symptoms included in physician studies (61). It is important to note that as our review included both clinically diagnosed depression and self-reported depressive symptoms, the pooled proportion is not directly comparable to global prevalence estimates that are based solely on diagnosed depression. Due to limitations of the current literature, a true estimate of the prevalence of depression among the profession remains uncertain.

While all studies included sex in the form of descriptive statistics, only six reported depression or depressive outcomes by sex. We found no statistically significant differences in male compared to female physicians (OR: 0.78; 95% CI: 0.46, 1.31); five studies showed that female physicians were more likely to be depressed or have depressive symptoms, while one study described the opposite trend (21, 29, 34, 40, 53, 58). Among the general population, females have a higher prevalence of depression than compared to males (62). Reasons for this disparity among physicians specifically may be a result of female physicians being less likely to report depression/depressive symptoms due to concerns regarding competency or career advancement, lack of time, or stigma (63).

When assessing resident physicians, we observed a slightly higher estimate of depression and depressive symptoms (36%, 95% CI: 26-47%) than compared to staff physicians (29%, 95% CI:13-53%). However, only two studies provided information on both resident and staff physicians, which found that staff physicians had lower odds of being depressed than residents (OR: 0.66, 95% CI: 0.22, 1.98) (23, 34). When following residents over the course of their training, Rahmati et al. and Campbell et al. found that residents were less likely to be depressed in the third year of residency compared to the first (20, 48). However, when comparing the same populations, Rosen et al. reported the opposite trend (33). Examining changes in depression and depressive symptoms across ages could provide insight into whether certain stages of medical training are associated with higher risk. However, studies that reported age-specific groups were scarce, with no clear discernable age periods identified as higher risk. Longitudinal studies that follow physicians throughout their careers would aid our understanding of the role of depression and depressive symptoms across ages. This is especially important to consider as research in the general population shows that older individuals with major depressive disorder often experience a worse disease course than younger individuals, suggesting the long-term impact of depression in physicians may be especially severe if symptoms continue into later career stages (60).

The rates of depression and depressive symptoms varied heavily between specialties, with the highest reports being amongst radiologists, surgeons, and internal medicine physicians. Commander et al. found that 70.5% of surgeons surveyed were depressed, while Dyrbye et al. found that 30.1% of surgeons were depressed (21, 54). This may be the result of studies using different assessment methods with varying cutoff scores, or due to differences in practicing location and underlying and stress factors. In addition, self-perceived stress, which serves as a key precursor to burnout and depression, is reported to vary considerably between physician specialties (50). Therefore, true differences in depression may exist and require studies with direct comparison between specialties to quantify. This information is important to the medical profession as the identification of high-risk specialties would facilitate directed screening and/or interventions to promote improved well-being.

Finally, stigma surrounding psychological distress and mental illness in healthcare is a significant consideration when assessing our findings. Physicians may be reluctant to acknowledge or report psychological distress (64–66). However, many of the included studies were conducted in anonymized settings, which may have encouraged more true reporting of depressive symptoms. Simultaneously, it must also be considered that over the nearly two-decade study period, there have been significant changes in attitudes toward mental health within the healthcare profession. Changes in increased awareness of mental health and decreased stigma may have influenced both the experience and reporting of depressive symptoms (67). These systematic changes may have contributed to variation in prevalence rates across included studies.

There were several limitations identified in the observed literature on depression and depressive symptoms prior to the COVID-19 pandemic. To begin, studies limited to distinct regions or specific hospitals may be influenced by cultural or institutional factors that are not generalizable to broader physician populations. Next, cross-sectional survey studies, which were the most common study design observed, are at increased risk for non-response bias. This may skew prevalence estimates if those experiencing depressive symptoms were either more or less likely to participate in studies. Next, the heterogeneity in cut-off scores limited the ability to pool or meta-analyze our findings, as variation in individual thresholds introduces uncertainty in comparisons across studies. In addition, the exclusion of non-English studies and grey literature may introduce both language and publication bias, thus leading to either an over- or underestimation of true prevalence. As a result, these findings should be interpreted with caution, as the prevalence estimates may not fully capture unpublished or non-English language data. Furthermore, the tools and definitions used were highly heterogenous, rendering cross comparisons difficult, especially when considering the high proportion of self-reported results. As such, the pooled prevalence estimate reflects both clinically diagnosed depression and self-reported depressive symptoms, based on how each study defined and measured the outcome, which may contribute to variability and limit comparability across studies. Additionally, the 18-year study period spans a time of significant cultural and systemic changes in healthcare, which may have influenced both the prevalence and reporting of depressive symptoms Finally, a limitation of our meta-analysis is the high degree of statistical heterogeneity observed across studies (I² = 98%). This degree of heterogeneity suggests substantial variability in the underlying data, which may be attributable to a variety of factors such as differences in study populations, geographic settings, measurement tools, and definitions of key variables. Although we report a pooled prevalence estimate to provide an overall effect measure, the presence of high heterogeneity limits the generalizability of the pooled estimate; thus, it is important to interpret this value with caution.

## 5 Conclusions

In this systematic review and meta-analysis examining depression and depressive symptoms among physicians, we report a pooled estimate of 34% prior to the COVID-19 pandemic. Most studies were cross-sectional and survey based, with a large degree of heterogeneity in the assessment tools used and defined cut-off scores. Studies lacked appropriate stratification and comparison by age, sex, physician specialty, and training stage, rendering the identification of key subgroups at risk difficult. The information presented in this review can serve as a baseline to compare changes that occurred in physician depression and depressive symptoms as a result of the COVID-19 pandemic. These findings underscore the need for targeted multifaceted interventions, including screening policies, workplace mental health initiatives, and support systems, uniquely tailored to physicians’ sex, stage of training, and specialty. Further population-based cohort studies with robust methodologies are required to determine the true prevalence of depression within physician populations.

## Data Availability

The original contributions presented in the study are included in the article/**Supplementary Material**. Further inquiries can be directed to the corresponding author.
