# Supplementary material for: Depression and depressive symptoms in physicians prior to the COVID-19 pandemic: a systematic review and meta-analysis
Source: Front Psychiatry. 2025 Oct 22;16:1627507. doi: 10.3389/fpsyt.2025.1627507 (PMC12586131; doi:10.3389/fpsyt.2025.1627507)
Supplement: Supplementary file 1 [file SupplementaryFile1.docx]

Supplementary Material

**Detailed Search Strategy**

**Modified Newcastle-Ottawa scale**

**PRISMA 2020 Checklist**

**Supplemental Figure 1.** Forest plot on the prevalence of depression/depressive symptoms by study design (cross sectional survey CSS or cohort study)

**Supplemental Figure 2:** Forest plot on the proportion of depression/depressive symptoms by physician specialty

**Supplemental Figure 3.** Forest plot on the proportion of depression/depressive symptoms by sex

**Supplemental Figure 4.** Forest plot on the proportion of depression/depressive symptoms among resident physicians

**Supplemental Figure 5.** Forest plot on the proportion of depression/depressive symptoms among fully trained, staff physicians

**Supplemental Figure 6.** Forest plot on the proportion of depression/depressive symptoms of studies directly comparing residents and fully trained, staff physicians.

**Supplemental Table 1.** Risk of Bias assessment of included studies using the modified Newcastle-Ottawa Scale presented by Mata et al. ^7^

**Detailed Search Strategy**: Completed November 19, 2020

Medline (OVID interface)

Date restriction: 2002-2020

| Physician Terms | Depression-Associated MeSH Terms |
| --- | --- |
| doctor*. ti, ab, kf. OR physician*. ti, ab, kf. OR resident*. ti, ab, kf. OR Physicians/ OR "Internship and Residency"/ OR Cardiologists/ OR Pulmonary Medicine/ OR Internal Medicine/ OR Pediatricians/ OR Gynecology/ or Obstetrics/ OR Orthopedic Surgeons/ or "Oral and Maxillofacial Surgeons"/ or Surgeons/ OR Psychiatry/ OR Dermatologists/ OR Endocrinologists/ OR Gastroenterologists/ OR Nephrologists/ OR Ophthalmology/ OR Pulmonologists/ OR Neurologists/ OR Radiologists/ OR Anesthesiologists/ OR Oncologists/ OR Neurosurgeons/ OR Allergists/ OR Physicians, Family/ OR Physicians, Emergency/ OR Pathologists/ OR Physiatrists/ OR Rheumatologists/ OR Urologists/ OR cardiologist*.ti,ab,kf. OR (Pulmonary adj Medicine). ti, ab, kf. OR (Internal adj Medicine). ti, ab, kf. OR Pediatrician*. ti, ab, kf. OR Gynecolog*. ti, ab, kf. OR Obstetrics*. ti, ab, kf. OR Surgeon*. ti, ab, kf. OR Psychiatr*. ti, ab, kf. OR Dermatologist*. ti, ab, kf. OR Endocrinologist*. ti, ab, kf. OR Gastroenterologist*. ti, ab, kf. OR Nephrologist*. ti, ab, kf. OR Ophthalmolog*. ti, ab, kf. OR Neurologist*. ti, ab, kf. OR Radiologist*. ti, ab, kf. OR Anesthesiologist*. ti, ab, kf. OR Oncologist*. ti, ab, kf. OR Neurosurgeon*. ti, ab, kf. OR Allergist*. ti, ab, kf. OR (Family adj Medicine). ti, ab, kf. OR Pathologist*. ti, ab, kf. OR Physiatrist*. ti, ab, kf. OR Rheumatologist*. ti, ab, kf. OR Urologist*. ti, ab, kf. | MESH Terms: Depression/ Depressive Disorder, Major  Keywords:  (depressi*). ti, ab, kf. OR (major adj1 depressive adj1 disorder*). ti, ab, kf. OR (involutional adj1 paraphrenia *). ti, ab, kf. OR (involutional adj1 psychos**). ti, ab, kf. |

APA PsychInfo (OVID interface)

Date restriction: 2002-2020 (manually screen from March 2020-December 2020)

| Physician Terms | Depression-Associated MeSH Terms |
| --- | --- |
| doctor*. ti, ab. OR physician*. ti, ab. OR residen*. ti, ab. OR Physicians/  OR "Internship and Residency"/ OR  Cardiologists/ OR Pulmonary Medicine/ OR Internal Medicine/ OR Pediatricians/ OR Gynecology/ or Obstetrics/ OR Orthopedic Surgeons/ or "Oral and Maxillofacial Surgeons"/ or Surgeons/ OR Psychiatry/ OR Dermatologists/ OR Endocrinologists/ OR Gastroenterologists/ OR Nephrologists/ OR Ophthalmology/ OR Pulmonologists/ OR Neurologists/ OR Radiologists/ OR Anesthesiologists/ OR Oncologists/ OR Neurosurgeons/ OR Allergists/ OR Physicians, Family/ OR Physicians, Emergency/ OR Pathologists/ OR Physiatrists/ OR Rheumatologists/ OR Urologists/ OR cardiologist*.ti,ab. OR (Pulmonary adj Medicine).ti,ab. OR (Internal adj Medicine).ti,ab. OR Pediatrician*.ti,ab. OR Gynecolog*.ti,ab. OR Obstetrics*.ti,ab. OR Surgeon*.ti,ab. OR Psychiatr*.ti,ab. OR Dermatologist*.ti,ab. OR Endocrinologist*.ti,ab. OR Gastroenterologist*.ti,ab. OR Nephrologist*.ti,ab. OR Ophthalmolog*.ti,ab. OR Neurologist*.ti,ab. OR Radiologist*.ti,ab. OR Anesthesiologist*.ti,ab. OR Oncologist*.ti,ab. OR Neurosurgeon*.ti,ab. OR Allergist*.ti,ab. OR (Family adj Medicine).ti,ab. OR Pathologist*.ti,ab. OR Physiatrist*.ti,ab. OR Rheumatologist*.ti,ab. OR Urologist*.ti,ab. | MESH Terms: Depression/ Depressive Disorder, Major  Keywords:  (depressi*). ti, ab. OR (major adj1 depressive adj1 disorder *). ti, ab. OR (involutional adj1 paraphrenia *). ti, ab. OR (involutional adj1 psychos *). ti, ab. |

EMBASE (OVID interface)

Date restriction: 2002-2020

| Physician Terms | Depression-Associated MeSH Terms |
| --- | --- |
| doctor*. ti, ab. OR physician*. ti, ab. OR residen*. ti, ab. OR Physicians/  OR "Internship and Residency"/ OR  Cardiologists/ OR Pulmonary Medicine/ OR Internal Medicine/ OR Pediatricians/ OR Gynecology/ or Obstetrics/ OR Orthopedic Surgeons/ or "Oral and Maxillofacial Surgeons"/ or Surgeons/ OR Psychiatry/ OR Dermatologists/ OR Endocrinologists/ OR Gastroenterologists/ OR Nephrologists/ OR Ophthalmology/ OR Pulmonologists/ OR Neurologists/ OR Radiologists/ OR Anesthesiologists/ OR Oncologists/ OR Neurosurgeons/ OR Allergists/ OR Physicians, Family/ OR Physicians, Emergency/ OR Pathologists/ OR Physiatrists/ OR Rheumatologists/ OR Urologists/ OR cardiologist*.ti,ab. OR (Pulmonary adj Medicine).ti,ab. OR (Internal adj Medicine).ti,ab. OR Pediatrician*.ti,ab. OR Gynecolog*.ti,ab. OR Obstetrics*.ti,ab. OR Surgeon*.ti,ab. OR Psychiatr*.ti,ab. OR Dermatologist*.ti,ab. OR Endocrinologist*.ti,ab. OR Gastroenterologist*.ti,ab. OR Nephrologist*.ti,ab. OR Ophthalmolog*.ti,ab. OR Neurologist*.ti,ab. OR Radiologist*.ti,ab. OR Anesthesiologist*.ti,ab. OR Oncologist*.ti,ab. OR Neurosurgeon*.ti,ab. OR Allergist*.ti,ab. OR (Family adj Medicine).ti,ab. OR Pathologist*.ti,ab. OR Physiatrist*.ti,ab. OR Rheumatologist*.ti,ab. OR Urologist*.ti,ab. | MESH Terms: Depression/ Depressive Disorder, Major  Keywords:  (depressi*). ti, ab. OR (major adj1 depressive adj1 disorder *). ti, ab. OR (involutional adj1 paraphrenia *). ti, ab. OR (involutional adj1 psychos *). ti, ab. |

Modified Newcastle-Ottawa scale taken from Mata et al. ^7^

1. Representativeness of the sample:

1 point: Population included had to be from (a) more than 1 specialty AND (b) more than 1 study center

0 point: Population was only taken from 1 specialty and study site.

1. Sample size:

- 1 point: Sample size greater than or equal to 200 physicians and/or residents
- 0 point: Sample size less than 200 physicians and/or residents, or if sample size greater than 200 but included medical students with no information provided on physicians or residents on their own

1. Non-respondents

- 1 point: Comparability between respondent and non-respondent characteristics was established, AND a response rate greater than 80%.
- 0 point: Insufficient or no information on the comparability between respondents and non-respondents, no response rate or response rate less than 80%, or no description of the characteristics of the responders and non-responders.

1. Ascertainment of depression:

- 1 point: Validated measure tool using a validated cutoff score
- 0 point: non-validated measurement tool, or validated measurement tool with non-valid cut-off score, or 2-item PRIME-MD

1. Quality of descriptive reporting:

- 1 point: Reported descriptive statistics to describe the population (ex. Age, sex) with proper measures of dispersion (ex. Standard deviation, standard error, range)
- 0 point: descriptive statistics were not reported, were incomplete, or did not include proper measures of dispersion.

Legend: A total score was given for each study (0-5) with studies that had a score >= 3 considered low risk of bias and a score <=2 a high risk of bias

**PRISMA – 2020**

| **Section and Topic** | **Item #** | **Checklist item** | **Location where item is reported** |
| --- | --- | --- | --- |
| **TITLE** | | | |
| Title | 1 | Identify the report as a systematic review. | Page 1 |
| **ABSTRACT** | | | |
| Abstract | 2 | See the PRISMA 2020 for Abstracts checklist. | Page 2 |
| **INTRODUCTION** | | | |
| Rationale | 3 | Describe the rationale for the review in the context of existing knowledge. | Page 4 |
| Objectives | 4 | Provide an explicit statement of the objective(s) or question(s) the review addresses. | Page 4 |
| **METHODS** | | | |
| Eligibility criteria | 5 | Specify the inclusion and exclusion criteria for the review and how studies were grouped for the syntheses. | Page 5 |
| Information sources | 6 | Specify all databases, registers, websites, organisations, reference lists and other sources searched or consulted to identify studies. Specify the date when each source was last searched or consulted. | Page 5 & 6 + Supplementary material |
| Search strategy | 7 | Present the full search strategies for all databases, registers and websites, including any filters and limits used. | Supplementary material |
| Selection process | 8 | Specify the methods used to decide whether a study met the inclusion criteria of the review, including how many reviewers screened each record and each report retrieved, whether they worked independently, and if applicable, details of automation tools used in the process. | Page 6 |
| Data collection process | 9 | Specify the methods used to collect data from reports, including how many reviewers collected data from each report, whether they worked independently, any processes for obtaining or confirming data from study investigators, and if applicable, details of automation tools used in the process. | Page 6 |
| Data items | 10a | List and define all outcomes for which data were sought. Specify whether all results that were compatible with each outcome domain in each study were sought (e.g. for all measures, time points, analyses), and if not, the methods used to decide which results to collect. | Page 6 |
|  | 10b | List and define all other variables for which data were sought (e.g. participant and intervention characteristics, funding sources). Describe any assumptions made about any missing or unclear information. | Page 6 |
| Study risk of bias assessment | 11 | Specify the methods used to assess risk of bias in the included studies, including details of the tool(s) used, how many reviewers assessed each study and whether they worked independently, and if applicable, details of automation tools used in the process. | Page 7 |
| Effect measures | 12 | Specify for each outcome the effect measure(s) (e.g. risk ratio, mean difference) used in the synthesis or presentation of results. | Page 7 |
| Synthesis methods | 13a | Describe the processes used to decide which studies were eligible for each synthesis (e.g. tabulating the study intervention characteristics and comparing against the planned groups for each synthesis (item #5)). | Page 7 |
|  | 13b | Describe any methods required to prepare the data for presentation or synthesis, such as handling of missing summary statistics, or data conversions. | Page 7 |
|  | 13c | Describe any methods used to tabulate or visually display results of individual studies and syntheses. | Page 7 |
|  | 13d | Describe any methods used to synthesize results and provide a rationale for the choice(s). If meta-analysis was performed, describe the model(s), method(s) to identify the presence and extent of statistical heterogeneity, and software package(s) used. | Page 7 |
|  | 13e | Describe any methods used to explore possible causes of heterogeneity among study results (e.g. subgroup analysis, meta-regression). | Page 7 |
|  | 13f | Describe any sensitivity analyses conducted to assess robustness of the synthesized results. | Page 7 |
| Reporting bias assessment | 14 | Describe any methods used to assess risk of bias due to missing results in a synthesis (arising from reporting biases). | Page 7 |
| Certainty assessment | 15 | Describe any methods used to assess certainty (or confidence) in the body of evidence for an outcome. | Page 7 |
| **RESULTS** | | | |
| Study selection | 16a | Describe the results of the search and selection process, from the number of records identified in the search to the number of studies included in the review, ideally using a flow diagram. | Page 7 |
|  | 16b | Cite studies that might appear to meet the inclusion criteria, but which were excluded, and explain why they were excluded. | Page 7 |
| Study characteristics | 17 | Cite each included study and present its characteristics. | Page 8 - 11 |
| Risk of bias in studies | 18 | Present assessments of risk of bias for each included study. | Page 11 |
| Results of individual studies | 19 | For all outcomes, present, for each study: (a) summary statistics for each group (where appropriate) and (b) an effect estimate and its precision (e.g. confidence/credible interval), ideally using structured tables or plots. | Page 8 - 11 |
| Results of syntheses | 20a | For each synthesis, briefly summarise the characteristics and risk of bias among 20contributing studies. | Page 8 - 11 |
|  | 20b | Present results of all statistical syntheses conducted. If meta-analysis was done, present for each the summary estimate and its precision (e.g. confidence/credible interval) and measures of statistical heterogeneity. If comparing groups, describe the direction of the effect. | Page 9 |
|  | 20c | Present results of all investigations of possible causes of heterogeneity among study results. | Page 8 - 11 |
|  | 20d | Present results of all sensitivity analyses conducted to assess the robustness of the synthesized results. | Page 8 - 11 |
| Reporting biases | 21 | Present assessments of risk of bias due to missing results (arising from reporting biases) for each synthesis assessed. | Page 11 |
| Certainty of evidence | 22 | Present assessments of certainty (or confidence) in the body of evidence for each outcome assessed. | Page 8 - 11 |
| **DISCUSSION** | | | |
| Discussion | 23a | Provide a general interpretation of the results in the context of other evidence. | Page 11 - 14 |
|  | 23b | Discuss any limitations of the evidence included in the review. | Page 14 |
|  | 23c | Discuss any limitations of the review processes used. | Page 14 |
|  | 23d | Discuss implications of the results for practice, policy, and future research. | Page 15 |
| **OTHER INFORMATION** | | | |
| Registration and protocol | 24a | Provide registration information for the review, including register name and registration number, or state that the review was not registered. | Page 3 and 5 |
|  | 24b | Indicate where the review protocol can be accessed, or state that a protocol was not prepared. | Page 3 and 5 |
|  | 24c | Describe and explain any amendments to information provided at registration or in the protocol. | N/A |
| Support | 25 | Describe sources of financial or non-financial support for the review, and the role of the funders or sponsors in the review. | Page 16 |
| Competing interests | 26 | Declare any competing interests of review authors. | Page 16 |
| Availability of data, code and other materials | 27 | Report which of the following are publicly available and where they can be found: template data collection forms; data extracted from included studies; data used for all analyses; analytic code; any other materials used in the review. | Page 16 + Supplementary Material |

*From:*  Page MJ, McKenzie JE, Bossuyt PM, Boutron I, Hoffmann TC, Mulrow CD, et al. The PRISMA 2020 statement: an updated guideline for reporting systematic reviews. BMJ 2021;372:n71. doi: 10.1136/bmj.n71. This work is licensed under CC BY 4.0. To view a copy of this license, visit <https://creativecommons.org/licenses/by/4.0/>

**Supplemental Figure 1.** Forest plot on the prevalence of depression/depressive symptoms by study design (cross sectional survey CSS or cohort study)

**
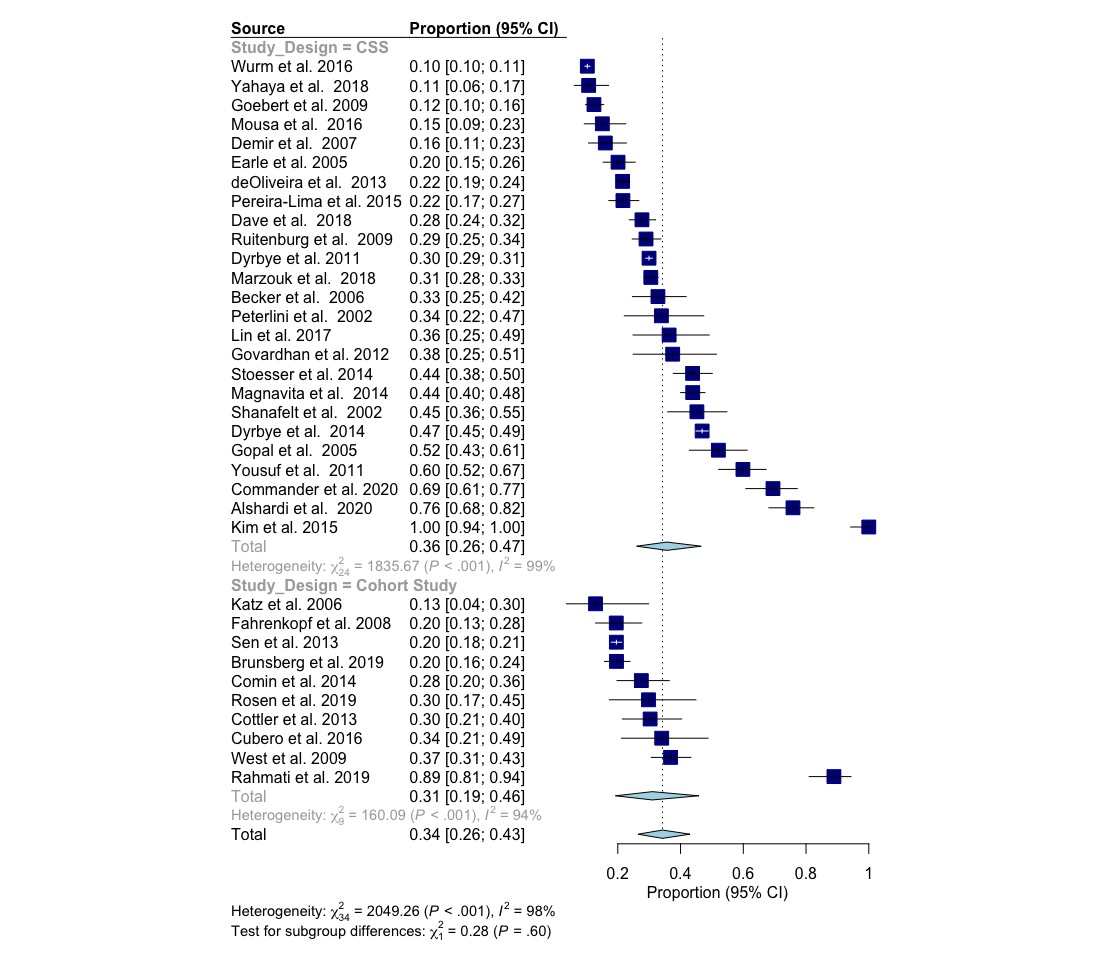
**

**Supplemental Figure 2:** Forest plot on the proportion of depression/depressive symptoms by physician specialty

**
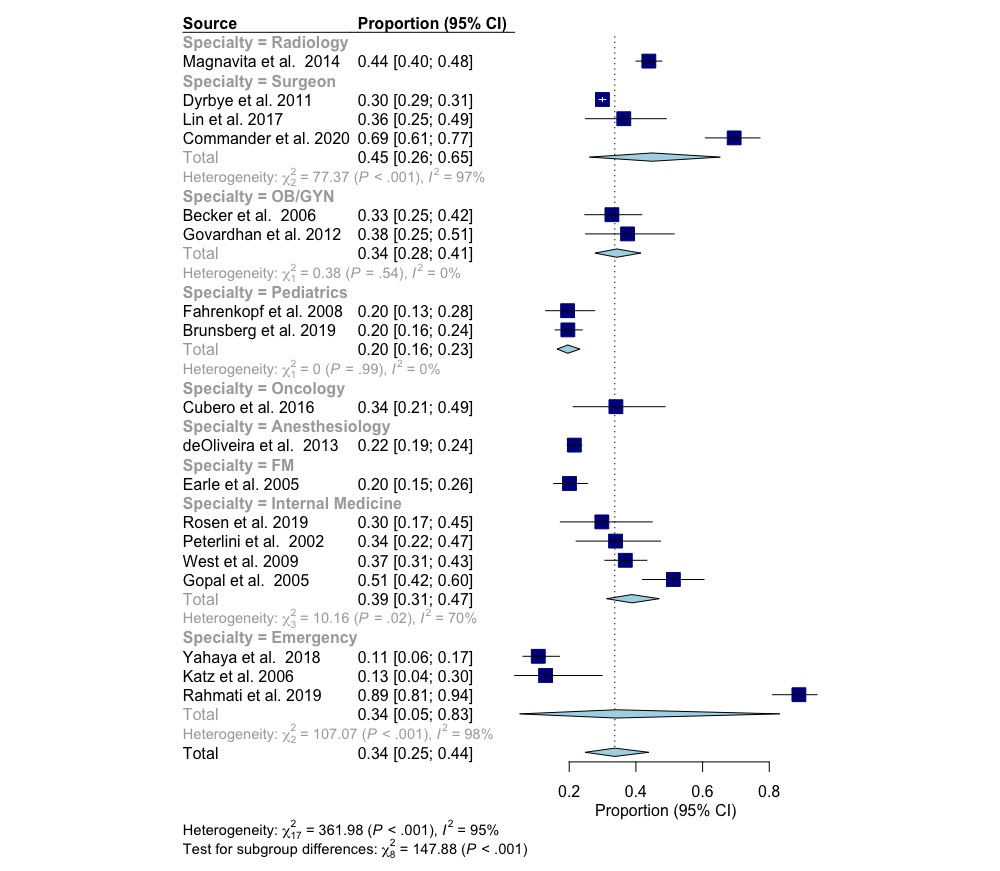
**

**Supplemental Figure 3.** Forest plot on the proportion of depression/depressive symptoms by sex


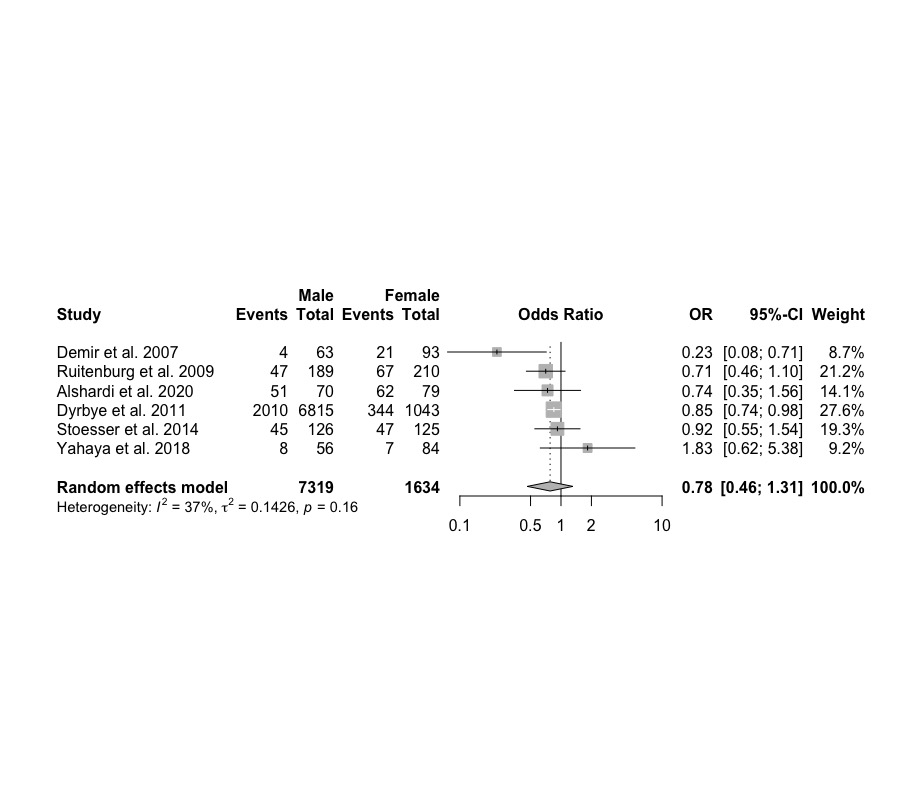


**Supplemental Figure 4.** Forest plot on the proportion of depression/depressive symptoms among resident physicians

**
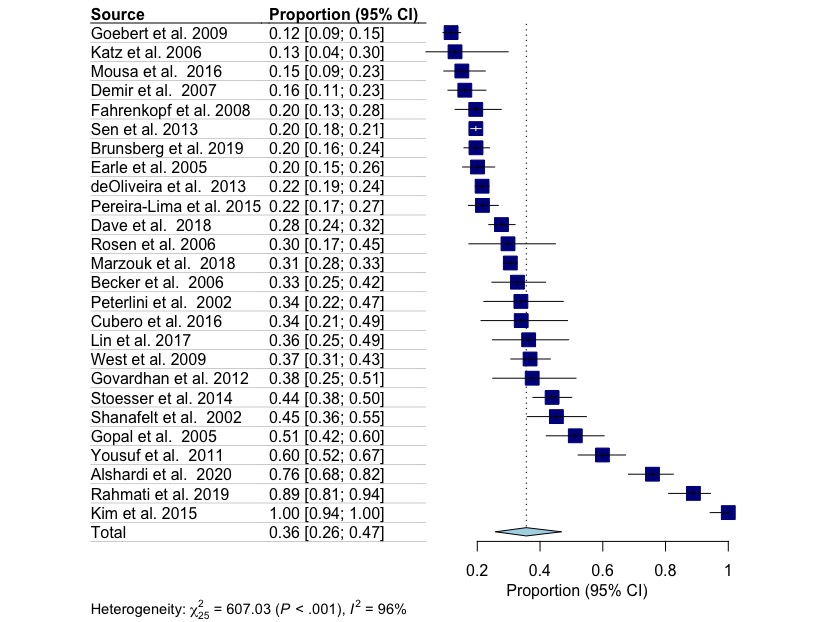
**

**Supplemental Figure 5.** Forest plot on the proportion of depression/depressive symptoms among fully trained, staff physicians


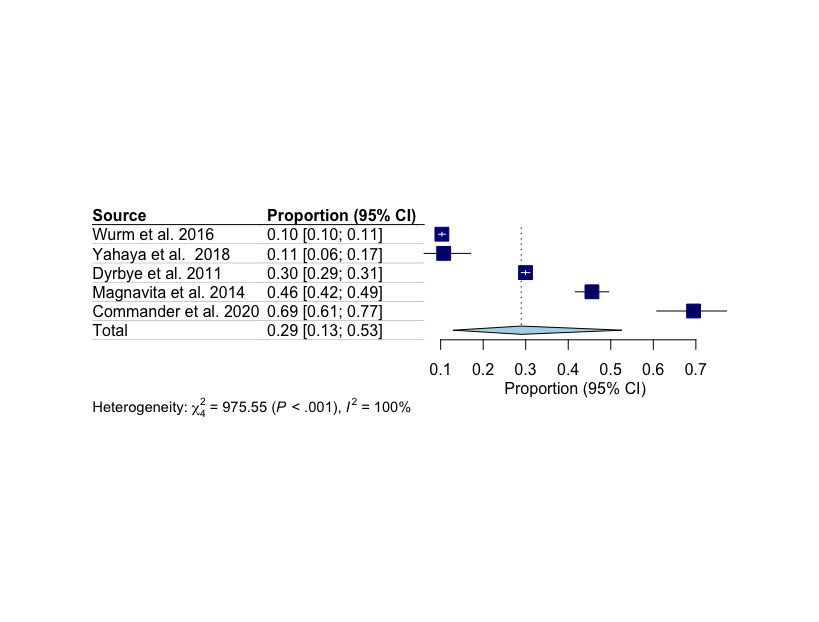


**Supplemental Figure 6.** Forest plot on the proportion of depression/depressive symptoms of studies directly comparing residents and fully trained, staff physicians.


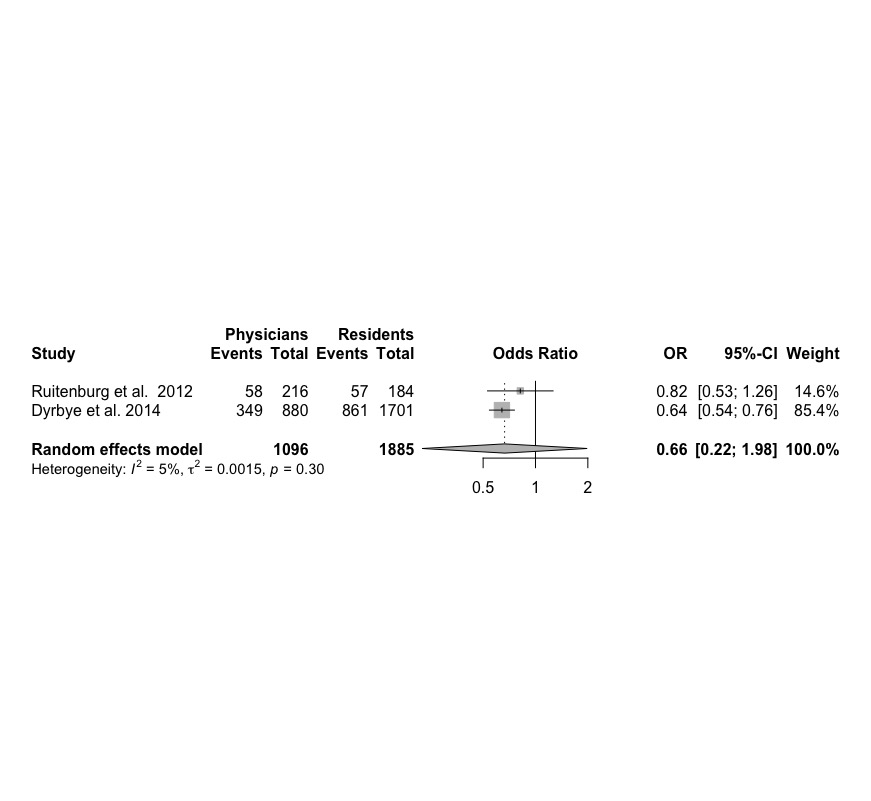


**Supplemental Table 1.** Risk of Bias assessment of included studies using the modified Newcastle-Ottawa Scale presented by Mata et al. ^7^

| Author, Year | Representativeness of the sample | Sample Size | Non-respondents | Ascertainment of depression | Quality of descriptive statistics reporting | Overall |
| --- | --- | --- | --- | --- | --- | --- |
| Alshardi et al. [30] 2020 | 0 | 0 | 0 | 1 | 1 | High |
| Balch et al. [18] 2011 | 1 | 1 | 0 | 0 | 1 | Low |
| Balch et al. [18] 2010 | 1 | 1 | 0 | 0 | 1 | Low |
| Becker et al. [47] 2006 | 0 | 0 | 0 | 1 | 1 | High |
| Brunsberg et al. [48] 2019 | 0 | 1 | 0 | 1 | 1 | Low |
| Campbell et al. [20] 2010 | 0 | 0 | 0 | 0 | 1 | High |
| Comin et al. [52] 2014 | 0 | 0 | 0 | 1 | 1 | High |
| Commander et al. [31] 2020 | 1 | 0 | 0 | 1 | 0 | High |
| Cottler et al. [59] 2013 | 1 | 0 | 0 | 1 | 1 | Low |
| Cubero et al. [38] 2016 | 0 | 0 | 1 | 0 | 1 | High |
| Dave et al. [54] 2018 | 0 | 1 | 0 | 1 | 0 | High |
| Demir et al. [37] 2007 | 0 | 0 | 0 | 1 | 1 | High |
| de Oliveira et al. [50] 2013 | 0 | 1 | 0 | 1 | 1 | Low |
| Dyrbye et al. [64] 2011 | 1 | 1 | 0 | 0 | 1 | Low |
| Dyrbye et al. [22] 2012 | 1 | 1 | 0 | 0 | 0 | High |
| Dyrbye et al. [23] 2014 | 1 | 1 | 0 | 0 | 0 | High |
| Earle et al. [36] 2005 | 0 | 1 | 0 | 1 | 0 | High |
| Fahrenkopf et al. [49] 2008 | 1 | 0 | 0 | 1 | 1 | Low |
| Goebert et al.[45] 2009 | 1 | 1 | 0 | 1 | 0 | Low |
| Gopal et al. [26] 2005 | 0 | 1 | 0 | 0 | 1 | High |
| Govardhan et al. [44] 2012 | 0 | 0 | 0 | 1 | 1 | High |
| Kalmbach et al. [32] 2017 | 1 | 1 | 0 | 1 | 1 | Low |
| Katz et al. [46] 2006 | 0 | 0 | 0 | 1 | 1 | High |
| Kim et al. [40] 2015 | 0 | 0 | 0 | 1 | 0 | High |
| Lin et al. [41] 2017 | 0 | 0 | 0 | 1 | 1 | High |
| Magnavita et al. [55] 2014 | 0 | 1 | 0 | 1 | 1 | Low |
| Marzouk et al. [56] 2018 | 0 | 1 | 0 | 1 | 1 | Low |
| Mousa et al. [28] 2016 | 0 | 0 | 0 | 1 | 0 | High |
| Pereira-Lima et al. [29] 2015 | 0 | 1 | 0 | 1 | 1 | Low |
| Peterlini et al. [39] 2002 | 0 | 0 | 0 | 1 | 0 | High |
| Rahmati et al. [58] 2019 | 0 | 0 | 0 | 1 | 1 | High |
| Ruitenburg et al. [43] 2012 | 0 | 1 | 0 | 1 | 1 | Low |
| Sen et al. [33] 2010 | 1 | 1 | 0 | 1 | 1 | Low |
| Sen et al. [34] 2013 | 1 | 1 | 0 | 1 | 0 | Low |
| Shanafelt et al. [27] 2002 | 0 | 0 | 0 | 0 | 0 | High |
| Stoesser et al. [35] 2014 | 0 | 1 | 0 | 1 | 1 | Low |
| West et al. [24] 2006 | 0 | 0 | 1 | 0 | 1 | High |
| West et al. [25] 2009 | 0 | 1 | 0 | 0 | 1 | High |
| Wurm et al. [57] 2016 | 1 | 1 | 0 | 1 | 1 | Low |
| Yahaya et al. [53] 2018 | 0 | 0 | 0 | 1 | 1 | High |
| Yousuf et al. [51] 2011 | 0 | 0 | 0 | 1 | 1 | High |

Studies were considered to have low risk of bias (>=3 points) or high risk of bias (<3 points).
